# Supplementary figures and images for: Characterization of the Pro-Inflammatory and Pruritogenic Transcriptome in Skin Lesions of the Experimental Canine Atopic Acute IgE-Mediated Late Phase Reactions Model and Correlation to Acute Skin Lesions of Human Atopic Dermatitis
Source: Vet Sci. 2024 Mar 1;11(3):109. doi: 10.3390/vetsci11030109 (PMC10974753; doi:10.3390/vetsci11030109)

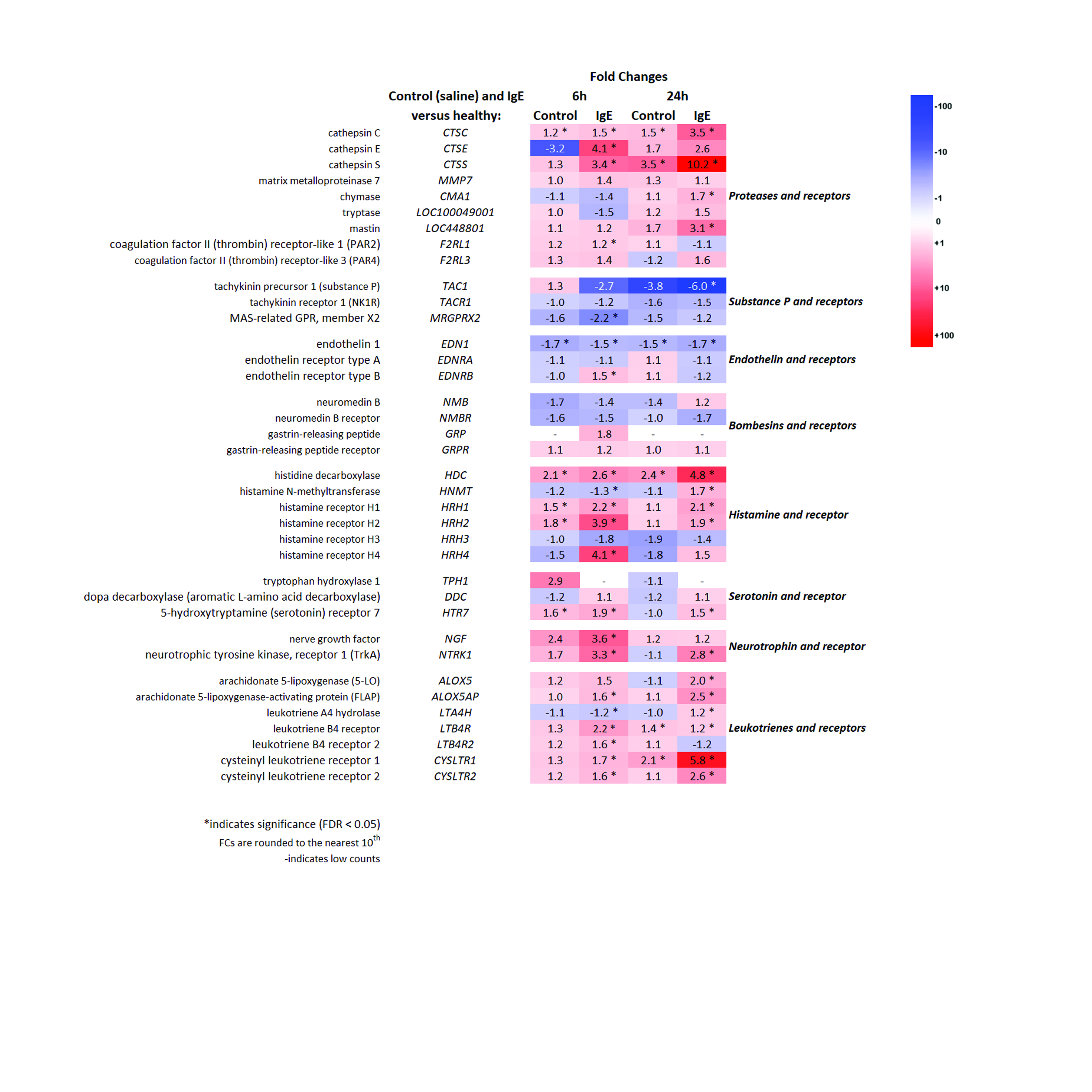

Supplement: Supplementary file 1 [file vetsci-11-00109-s001.zip › All figure and suppl files/Supplementary Figure_2_IgE_ver0822_pruritogens_heatmap.tif]
